# An Ultrafast *N-*Glycoproteome Analysis Method Using Thermoresponsive Magnetic Fluid-Immobilized Enzymes

**DOI:** 10.3389/fchem.2021.676100

**Published:** 2021-04-26

**Authors:** Zhiya Fan, Tong Liu, Fei Zheng, Weijie Qin, Xiaohong Qian

**Affiliations:** ^1^State Key Laboratory of Proteomics, National Center for Protein Sciences (Beijing), Beijing Proteome Research Center, Beijing, China; ^2^College of Basic Medicine, Anhui Medical University, Hefei, China

**Keywords:** urine proteomics, protein glycosylation, immobilized enzyme, thermo-responsiveness, magnetic fluid

## Abstract

*N-*Glycosylation is one of the most common and important post-translational modification methods, and it plays a vital role in controlling many biological processes. Increasing discovery of abnormal alterations in *N-*linked glycans associated with many diseases leads to greater demands for rapid and efficient *N-*glycosylation profiling in large-scale clinical samples. In the workflow of global *N-*glycosylation analysis, enzymatic digestion is the main rate-limiting step, and it includes both protease digestion and peptide-*N*4–(*N*-acetyl-beta-glucosaminyl) asparagine amidase (PNGase) F deglycosylation. Prolonged incubation time is generally required because of the limited digestion efficiency of the conventional in-solution digestion method. Here, we propose novel thermoresponsive magnetic fluid (TMF)-immobilized enzymes (trypsin or PNGase F) for ultrafast and highly efficient proteome digestion and deglycosylation. Unlike other magnetic material-immobilized enzymes, TMF-immobilized enzymes display a unique temperature-triggered magnetic response behavior. At room temperature, a TMF-immobilized enzyme completely dissolves in an aqueous solution and forms a homogeneous system with a protein/peptide sample for efficient digestion but cannot be separated by magnetic force because of its excellent water dispersity. Above its lower critical solution temperature (LCST), thermoflocculation of a TMF-immobilized enzyme allows it to be easily recovered by increasing the temperature and magnetic force. Taking advantage of the unique homogeneous reaction of a TMF-immobilized enzyme, both protein digestion and glycopeptide deglycosylation can be finished within 3 min, and the whole sample processing time can be reduced by more than 20 times. The application of a TMF-immobilized enzyme in large-scale profiling of protein *N-*glycosylation in urine samples led to the successful identification of 2,197 *N*-glycopeptides and further demonstrated the potential of this strategy for fast and high-throughput analysis of *N-*glycoproteome in clinical samples.

## Introduction

Glycosylation is one of the most prominent post-translational modification methods for proteins (Stadlmann et al., 2017; Huang et al., 2019). As a major type, *N-*glycosylation has a wide range of functions that greatly amplifies the diversity of proteins (Hart and Copeland, 2010; Yang et al., 2020). From the general biological process, such as cell adhesion and signal transduction, to specific functions of proteins like folding and stability, the complexity imparted to a proteome by *N-*glycosylation is immense (Schjoldager et al., 2020). Moreover, the *N-*glycans biosynthesis process is very sensitive to the physiological and pathological states in cells (Mereiter et al., 2019; Dong et al., 2020), and glycoproteins are a main type of current therapeutic targets and disease biomarkers (Pan et al., 2020; Zhao et al., 2020). Fueled by the increasing discovery of disease-related *N-*glycans alterations, the interest in large-scale *N*-glycosylation profiling in clinical samples is proliferating. Urine, as a reflection of body changes, is considered as an ideal source for biomarker discovery (Wu and Gao, 2015). It can be obtained in a non-invasive manner with a relatively narrower protein dynamic range and much less interference by high-abundance proteins compared with that of plasma/serum (Zhao et al., 2017). Therefore, rapid and in-depth analysis of *N*-glycoproteome from a large cohort of urine samples is highly desirable for a clinical study.

In mass spectrometry-based shotgun *N*-glycoproteome analysis, enzymatic digestion of proteins and release of *N*-glycans are regarded as key steps, which are generally performed using a solution. However, because only a small amount of enzyme is used and digestion efficiency is limited in solution-based digestion, prolonged incubation time is needed, which highly limits sample throughput (Qin et al., 2012). To address these problems, various kinds of the immobilized enzyme have been developed with the advantages of reducing enzyme self-digestion, stabilizing enzymatic activity, and allowing higher enzyme concentration for shorter digestion time (Mateo et al., 2007). In contrast with conventional in-solution digestion, an immobilized enzyme for *in situ* digestion can be easily separated from the digestion system and reused. Various supports have been proven to be feasible for enzyme immobilization, such as nanomaterials (Sharifi et al., 2020), porous silicon matrices (Létant et al., 2004), porous polymer monoliths (Krenkova et al., 2009), sol-gel supports (Yuce-Dursun et al., 2016), membranes (Luo et al., 2014), magnetic beads (Zhao et al., 2015; Fauser et al., 2020), and graphene oxide (Yuan et al., 2017). Although the required digestion time is obviously decreased using these reagents, because of the insolubility of reported supporting materials, digestion is processed under solid–liquid heterogeneous conditions that may limit reaction efficiency. Therefore, a new immobilized enzyme with homogeneous digestion and heterogeneous separation characteristics has high demand.

**Figure 1:**
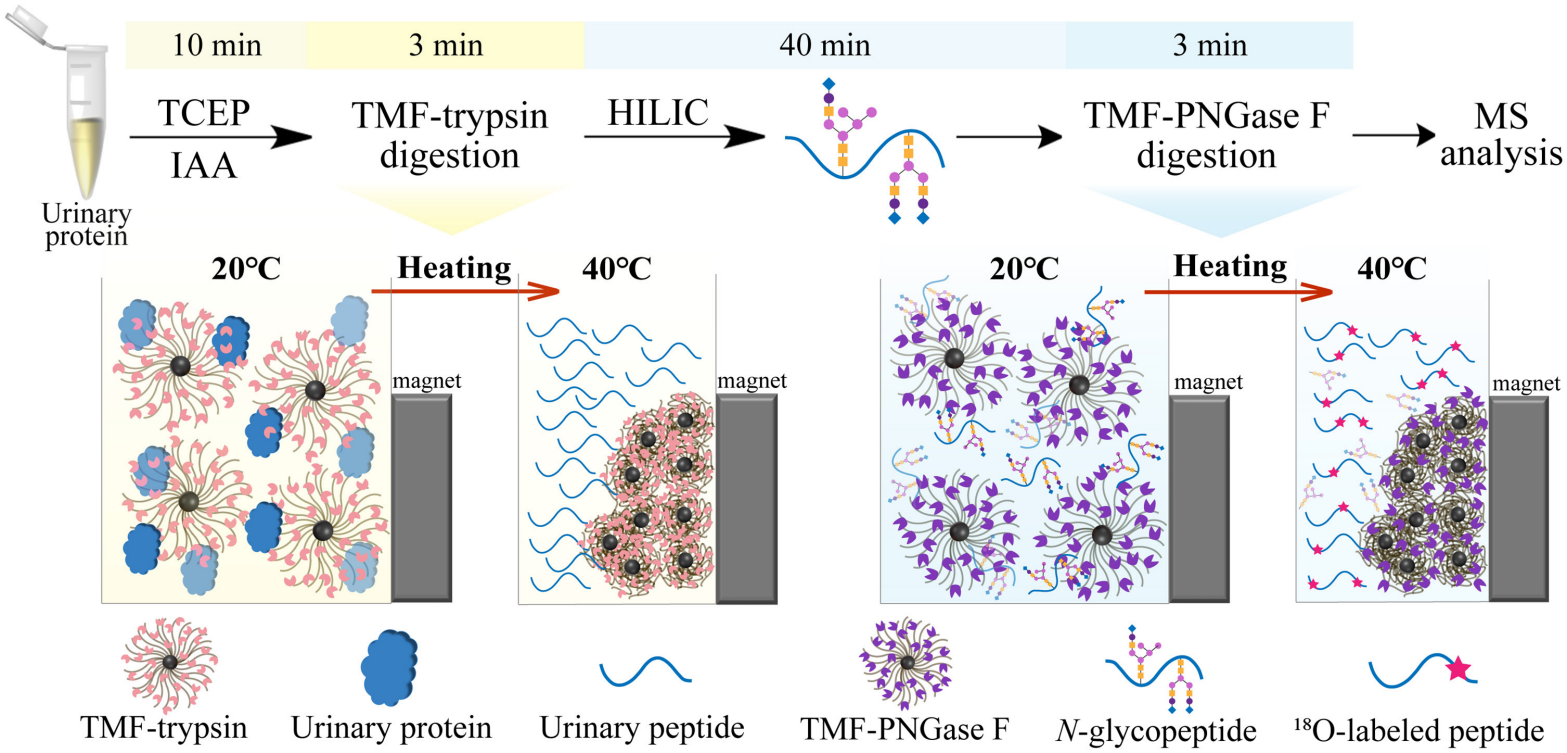
Workflow of ultrafast *N-*glycoprotein analysis by TMF–trypsin digestion and TMF–PNGase F deglycosylation.

In this study, we developed a new type of immobilized enzyme reagent based on thermoresponsive magnetic fluid (TMF) with a lower critical solution temperature (LCST) in an aqueous solution (Supplementary Figure 1). To the best of our knowledge, this is the first study to immobilize trypsin or PNGase F in TMF. TMF is composed of thermoresponsive polymer chains grafted from the surface of a small magnetic Fe_3_O_4_ core (~10 nm), *via in situ* growth using the surface-initiated atom transfer radical polymerization (SI-ATRP) technique. Thermoresponsive polymer chains are copolymerized using *N-*isopropylacrylamide (NIPAM) (Zhang et al., 2017) and undecylenic aldehyde (UnAl), which provides TMF with thermoresponsive property, water solubility, and reactivity with enzymes. Unlike other magnetic material-immobilized enzymes, TMF-immobilized enzymes exhibit a unique temperature-triggered magnetic response behavior. When the temperature is lower than LCST, the TMF-immobilized enzyme completely dissolves in an aqueous solution, and digestion is performed under homogeneous conditions, which therefore avoids interfacial mass transfer resistance in the two-phase heterogeneous system, and enhances enzyme–substrate interaction and reaction efficiency. After digestion and raising the temperature above LCST, the TMF-immobilized enzyme can be easily recovered by heat-triggered polymer thermoflocculation and magnetic separation (see Supplementary Video 1). Taking advantage of homogeneous reaction and high enzyme loading, either protein digestion or glycopeptide deglycosylation can be finished within 3 min, and the whole sample processing time can be reduced by more than 20 times (Figure 1) with digestion efficiency comparable to that of conventional in-solution overnight digestion. The successful application of TMF-immobilized enzymes in large-scale *N-*glycosylation identification using complex protein samples further demonstrated the potential of this strategy for high-throughput analysis of *N-*glycoproteome in clinical samples.

## Materials and Methods

### Preparation of TMF-Immobilized Enzyme

#### Synthesis of Oleic Acid-Coated Magnetic Nanoparticles (OA-Coated MNPs)

Oleic acid (OA)-coated magnetic Fe_3_O_4_ nanoparticles were synthesized according to the previously reported method (Sun et al., 2006) with minor modifications. FeSO_4_·7H_2_O (1.18 g, 4.24 mmol) and FeCl_3_·6H_2_O (2.05 g, 7.58 mmol) were dissolved in 50 ml deionized water with vigorous stirring. The solution was heated at 60°C, and then 25 ml 25% (w/w) NH_3_·H_2_O was added dropwise. The solution color changed from orange to black, leading to a black precipitate. Then, 0.5 ml OA was added dropwise into the dispersion slowly at 80°C for 1 h. The whole process was carried out in a nitrogen atmosphere. Next, magnetic nanoparticles were extracted from water into toluene. Water dispersion 50 and 50 ml toluene was mixed in a 250-ml extractor. After adding a small amount of NaCl, magnetic nanoparticles transferred into the toluene phase with good dispersity under the coating of OA. Finally, the toluene dispersion was evaporated to remove the solvent under reduced pressure and dried under vacuum. The content of OA-coated MNPs was redissolved into toluene to 10 mg/ml.

#### Synthesis of Initiator

As an initiator, 3-(2-bromoisobutyramido)propyl(triethoxy)–silane (BIBAPTES) was synthesized as follows: α-bromoisobutyryl bromide (0.1 ml, 0.8 mmol) was added dropwise to a cold solution of 3-aminopropyltriethoxysilane (ATEPS) (0.18 ml, 0.8 mmol) in dry toluene (10 ml) with triethylamine (TEA) (0.12 ml, 0.8 mmol) at 0°C. The mixture was magnetically stirred for 3 h at 0°C and further stirred for 10 h at room temperature to complete the reaction. The precipitate (triethylammonium bromide) was filtered off. The filtrate was evaporated under reduced pressure to remove unreacted TEA and dried under vacuum.

#### Synthesis of Initiator-Grafted Magnetic Nanoparticles

Initiator-grafted magnetic nanoparticles were synthesized by ligand exchange reaction: BIBAPTES (0.05 ml), TEA (1 ml), and OA-coated MNPs (50 mg) were added into 15 ml dry toluene in a nitrogen atmosphere. The mixture was stirred for 48 h at room temperature. Next, the mixture was centrifuged to remove salts (triethylammonium oleate). The supernatant was centrifuged at 20,000 *g* for 60 min, washed three times with toluene to remove un-grafted initiators and unreacted TEA, and dried under vacuum.

#### Synthesis of PNIPAM-b-PUnAl via Surface Initiated ATRP on Magnetic Nanoparticles

Initiator-grafted magnetic nanoparticles 60 mg were resuspended in 20 ml isopropanol/water (v/v: 3/1), and then *N*-isopropylacrylamide (NIPAM) (1 g, 8.85 mmol), purified Cu(I)Br (240 mg, 1.68 mmol), and Tris (2-dimethylaminoethyl)amine (Me_6_TREN) (0.44 ml, 1.68 mmol) were added in a nitrogen atmosphere. The mixture was agitated at room temperature for 6–8 h, and then UnAl (1.4 ml, 7.08 mmol) was added and reacted for a further 12–18 h at room temperature. Finally, the magnetic fluid with thermoresponsive property and aldehyde groups was obtained after dialysis for 48 h.

#### Trypsin or PNGase F Immobilization on TMF and Measurement of Enzyme Loading Capacity

Trypsin (or PNGase F) was immobilized on the PNIPAM-*b*-PUnAl-grafted magnetic nanoparticles *via* Borch reduction between the aldehyde groups of the copolymer chains and the free amino groups of trypsin (or PNGase F). Typically, 8 mg modified magnetic nanoparticles were dispersed in 1 ml PBS (pH 7.4) containing 1 mg trypsin (or 1,500 UPNGase F) and 5 mg NaBH_3_CN. Next, the mixture was agitated at 4°C for 12 h. After the reaction, the mixture was heated to 32°C to allow the magnetic nanoparticles to flocculate, and trypsin (or PNGase F)-attached magnetic nanoparticles were then collected with a magnet. The collected magnetic nanoparticles were washed three times by adding a 1 ml 50 mM NH_4_HCO_3_ aqueous solution and repeating dispersion and flocculation. The enzyme loading capability of TMF was determined by HPLC *via* calculation of the difference in the peak area of the bovine serum albumin (BSA) solution before and after immobilization, which is similar to that of trypsin or PNGase F.

### TMF–Trypsin or Free Trypsin Digestion of Standard Protein and Urinary Proteins

This study was approved by the Institutional Review Board of the Tianjin Baodi Hospital, and all donors approved the use of their urine samples. Standard protein (BSA) was dissolved in 50 mM ammonium bicarbonate (ABC, pH = 8). Mid-stream samples of morning urine were collected and centrifuged at 12,000 g and 4°C for 30 min to remove cell debris. The urinary protein was precipitated by ice-cooled acetone and dissolved in a lysis buffer (8 M urea, 100 mM Tris-HCl, pH = 8) to a concentration of 1 mg/ml. Each sample was heated for 10 min at 95°C to denature. Then, TCEP (10 mM) reduction and CAA (40 mM) alkylation were performed. For TMF–trypsin digestion, 1 mL of the protein solution was mixed with TMF–trypsin and incubated for 3 min at room temperature. After digestion, the immobilized trypsin was retained by a heating-magnet process, and the supernatant was collected for mass spectrometry analysis. The TMF–trypsin materials can be reused after washing them three times with 50 mM ABC. For free trypsin digestion, first, the protein solution was diluted to reduce the urea concentration to 1 M. Then, free trypsin was introduced into the denatured protein solution at a substrate to enzyme ratio (w/w) of 50:1 and incubated at 37°C for 16 h. After digestion, 2 μl of formic acid was added to terminate the reaction, and the supernatant was collected for mass spectrometry analysis.

### HILIC Enrichment of Urinary *N*-Glycopeptides

Zwitterionic hydrophilic interaction liquid chromatography (ZIC-HILIC) materials (5 mg) were washed with a 200 μl binding buffer (80% ACN, 1% TFA) three times and incubated with 80 μg urinary peptides (TMF–trypsin digestion products) for 0.5 h. After that, a 600 μl binding buffer was used to remove non-specifically absorbed peptides. Finally, the *N*-glycopeptides were eluted using a 200 μl elution buffer (0.1% FA) and then vacuum-dried.

### TMF–PNGase F or Free PNGase F Deglycosylation of Standard Glycoproteins and Urinary Glycopeptides

Standard glycoprotein RNase B (10 μg/μl) was dissolved in deionized water and heated at 95°C for 10 min to denature. Then, TCEP (10 mM) reduction and CAA (40 mM) alkylation were performed. Urinary *N-*glycopeptides were obtained according to *HILIC Enrichment of Urinary N-Glycopeptides* and dissolved in ${\text{H}}_{2}^{18}$O. For TMF–PNGase F deglycosylation, 4 μl of the protein solution was mixed with 1 ml TMF–PNGase F and incubated for 3 min at room temperature. The immobilized PNGase F was retained by a heating-magnet process, and the supernatant was collected for mass spectrometry analysis. The TMF–PNGase F materials can be reused after washing them three times with deionized water (or ${\text{H}}_{2}^{18}$O). For in-solution deglycosylation, PNGase F was introduced to the protein solution at a substrate to enzyme ratio (w/w) of 10:1 and incubated at 37°C for 16 h. No further pre-process was required for in-solution digestion before mass spectrometry analysis.

### Mass Spectrometry Analysis

#### MALDI-TOF MS

For (glyco)protein analysis, equivalent volumes of the protein solution, aqueous 2% trifluoroacetic acid (TFA), and a 2,5-dihydroxyacetophenone (2, 5-DHAP) matrix were mixed by pipetting up and down until the liquid became cloudy. The mixture (1 μl) was applied onto a matrix-assisted laser desorption ionization (MALDI) plate and air-dried. For tryptic peptides and glycopeptides analysis, equivalent volumes of the peptide solution and a 2,4-dihydroxybenzoic (DHB) matrix were mixed, deposited onto a MALDI plate (1 μl), and air-dried. A MALDI-time of flight (TOF) mass spectrometry (MS) instrument (Bruker, Bremen, Germany) performed in positive ion mode with a nitrogen pulsed laser (337 nm) was used for measurements.

#### LC-MS/MS

The resulting peptide mixture was analyzed using an Orbitrap Fusion Lumos Tribrid mass spectrometer coupled with an EASY-nLC 1,000 nano-LC system (ThermoFisher Scientific, Waltham, MA, USA). Peptide separation was performed on a 15-cm length reverse phase C_18_ column (150 nm id, 1.9 μm, 100 Å) using A and B buffers (buffer A:0.1% formic acid in water; buffer B:0.1% formic acid in acetonitrile) at a constant flow rate of 600 nl min^−1^. The gradient was set as follows: 7–15% B for 7 min, 15–25% B for 37 min, 25–40% B for 20 min, and 40–100% B for 7 min. The dynamic exclusion duration of data-dependent MS2 acquisition (DDA) is 18 s. For MS1 scan, mass spectra were acquired in the positive-ion mode over the range of 300–1,400 m/z with a resolution of 120,000 and a maximum ion injection time of 50 ms. MS2 spectra were acquired with an automatic gain control target value of 5.e3 and a maximum injection time of 35 ms with higher-energy collision dissociation (HCD) with a normalized collision energy of 30%.

### Data Processing

MALDI-TOF-MS spectra were analyzed using the FlexAnalysis software (version 3.4) to extract peaks and corresponding intensities. Then, Mascot search and sequence coverage calculation by allowing two missed cleavages were performed. LC-MS/MS raw data were analyzed using the MaxQuant software (version 1.6.17.0) to search against the UniPort Human database (updated on July 21, 2015). The main parameters were set as follows: (1) digestion mode was set as trypsin up to two missed cleavages allowed, (2) mass tolerances were 20 ppm and 4.5 ppm for the first search and main search, respectively, (3) fixed modification, carbamidomethyl (C), (4) variable modification, deamidation 18O (N), acetyl (protein N-term), oxidation (M), (5) false discovery rate (FDR) was set as ≤ 0.01 at the spectra, protein and modification levels, and (6) minimum and delta scores for the modified peptides were set as ≥40 and ≥6, respectively. For *N*-glycopeptides identification, a motif filter of NXT/S/C (where X cannot be P) in peptide sequence and a localization probability filter [deamidation 18O (N)] of ≥0.75 were applied.

### Characterization of TMF Materials

Dynamic laser scattering (DLS) characterization was performed using a DynaPro NanoStar instrument (Wyatt Technology, Sta. Barbara, CA, USA). Fourier transform-infrared (FT-IR) spectra were obtained using a Tensor 27 FT-IR spectrometer (Bruker Corporation, Billerica, MA, USA). Vibrating sample magnetometry (VSM) was performed using a PPMS-9 instrument (Quantum Design, San Diego, CA, USA). Thermogravimetric analysis (TGA) was performed using a SDT Q500 instrument (TA Instruments, New Castle, DE, USA).

## Results and Discussion

### Preparation and Characterization of TMF

TMF was prepared *via* surface-initiated atom transfer radical polymerization (SI-ATRP) of the Fe_3_O_4_ nanoparticles (Supplementary Figure 1). First, the OA-coated Fe_3_O_4_ nanoparticles were synthesized according to the method, and then the initiators were immobilized on the surface of Fe_3_O_4_ nanoparticles through ligand exchange reaction. In SI-ATRP modification, NIPAM and UnAl were sequentially added into the reaction system to provide thermoresponsive property and aldenyde for enzyme conjugation. As a result, the polymer shell thickness, LCST, and the amounts of reactive groups can be well-defined by controlling the amounts and polymerization time of each monomer. After SI-ATRP modification, a multilayer enzyme reagent can be easily obtained by attaching enzymes on the side chains of the copolymer *via* the formation of covalent bonds with the aldehyde groups provided by poly-UnAl. More importantly, the poly-NIPAM part not only enables TMF well-dispersity in water to become magnetic fluid, which allows the digestion process under homogeneous conditions, but also offers a temperature-triggered method to separate the TMF-immobilized enzyme from the digestion medium. TMF was characterized by dynamic light scattering (DLS), FT-IR spectroscopy, VSM, and thermogravimetric analysis (TGA) to confirm the successful growth of PNIPAM-*b*-PUnAl copolymer chains on the surface of the magnetic nanoparticles and determine the content of the grafted copolymer chains.

**Figure 2:**
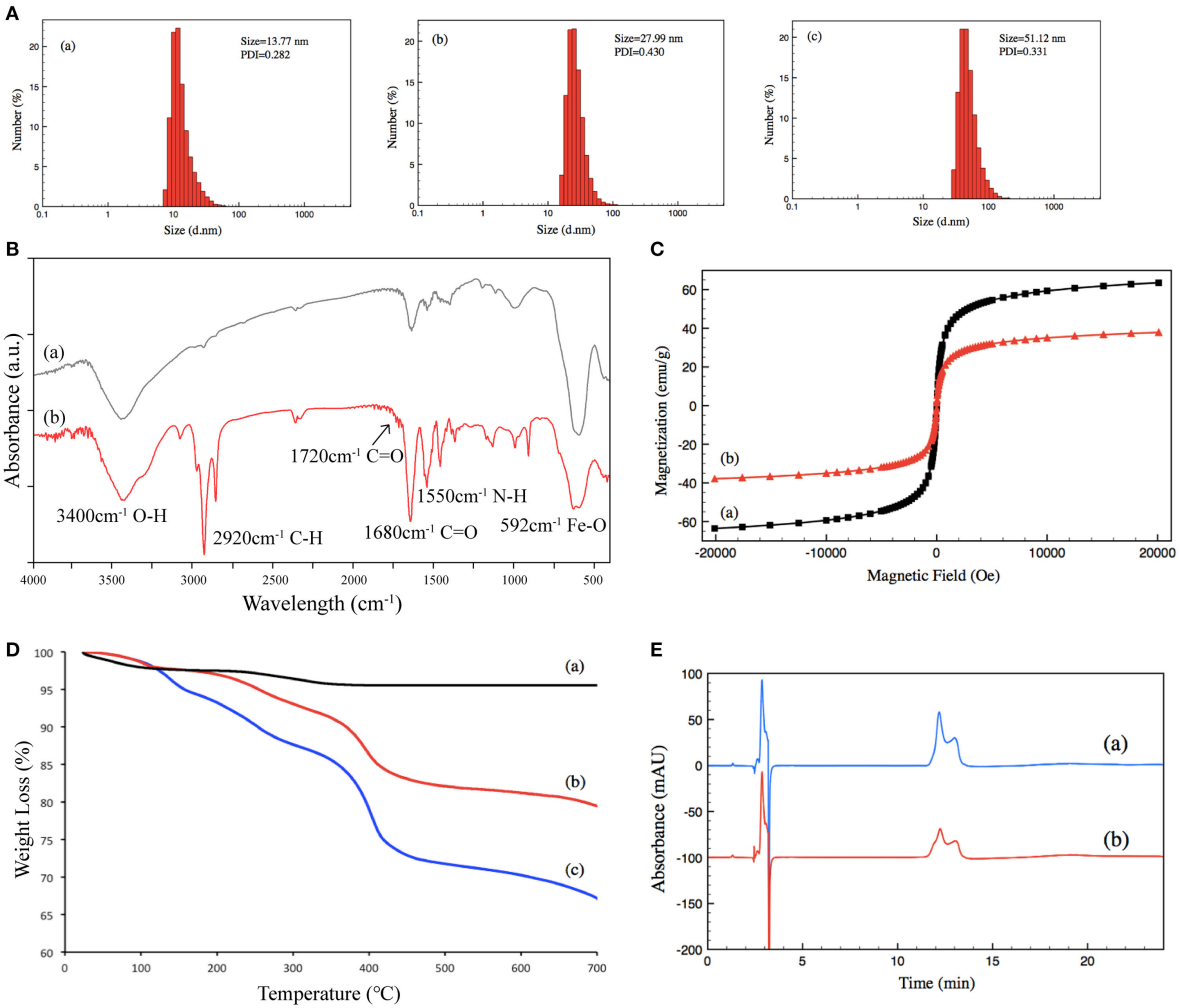
**(A)** Size distribution of (a) un-grafted magnetic nanoparticles, (b) poly-NIPAM grafted magnetic nanoparticles, and (c) poly-NIPAM*-b*-poly-UnAl copolymer-grafted magnetic nanoparticles. **(B)** FT-IR spectra of (a) un-grafted magnetic nanoparticles and (b) poly-NIPAM-*b*-poly-UnAl copolymer-grafted magnetic nanoparticles. **(C)** Magnetization curves at 25°C for (a) un-grafted magnetic nanoparticles and (b) poly-NIPAM*-b*-poly-UnAl copolymer-grafted magnetic nanoparticles. **(D)** TGA curves of (a) un-grafted magnetic nanoparticles, (b) poly-NIPAM grafted magnetic nanoparticles, and (c) poly-NIPAM-*b-*poly-UnAl copolymer-grafted magnetic nanoparticles. **(E)** HPLC spectra of (a) 0.5 μg/μl BSA and (b) supernatant solution after the immobilization of BSA on poly-NIPAM-*b*-poly-UnAl copolymer-grafted magnetic nanoparticles.

Size and size distribution were characterized by DLS at 25°C. The size of (a) un-grafted magnetic nanoparticles was 13.77 nm, (b) that of Poly-NIPAM grafted magnetic nanoparticles was 27.99 nm, and (c) that of PNIPAM-*b*-PUnAl copolymer grafted magnetic nanoparticles was 51.12 nm. Their PDI were 0.282, 0.430, and 0.331, respectively (Figure 2A). The increase in size is caused by the grafting of poly-NIPAM and poly-UnAlon magnetic nanoparticles. FT-IR was performed on (a) un-grafted magnetic nanoparticles and (b) PNIPAM-*b*-PUnAl copolymer grafted magnetic nanoparticles (Figure 2B). The peaks at 592 cm^−1^ of both (a) and (b) are assigned to the Fe–O bonds of the Fe_3_O_4_ core. Two relatively strong absorption peaks at 1,550 and 1,680 cm^−1^ corresponding to the N–H and C=O bonds in the amide groups of (b) indicate successful grafting of Poly-NIPAM on magnetic nanoparticles. A relatively weak absorption peak at 1,720 cm^−1^ ascribed to the C=O bonds in the aldehyde group indicates successful growth of poly-UnAl on magnetic nanoparticles. A strong absorption peak at 2,920 cm^−1^ ascribed to the C–H bond in the methylene group is observed in (b), which indicates that the polymer continuously grows on the surface of magnetic nanoparticles. Next, magnetic hysteresis loops for the magnetic Fe_3_O_4_ nanoparticles before and after grafting with the poly-NIPAM-*b-*poly-UnAl copolymer were determined by VSM testing (Figure 2C). The saturated magnetization (Ms) of un-grafted magnetic nanoparticles is 63.5 emu/g and decreased to 37.8 emu/g after the SI-ATRP with NIPAM and UnAl, because of the introduction of the un-magnetic copolymer. TGA analysis was carried out to further confirm the successful preparation of TMF as well as determine the content of surface-grafted polymer chains (Figure 2D). After thermo treatment over 700°C, the thermally stable Fe_3_O_4_ core remains in the residue and decomposable polymer chains contribute to weight loss. Unmodified magnetic nanoparticles (a) show <5% of total weight loss that might be attributed to the loss in water residue. In contrast, due to the decomposition of their polymer shell, modified magnetic nanoparticles showed noticeable weight loss. The weight loss is 21.7% for the magnetic nanoparticles with poly-NIPAM grafting (b) and increases to 32.85% after subsequent copolymerization with poly-UnAl (c), which indicates successful grafting of poly-NIPAM and poly-UnAl parts on the surface of the Fe_3_O_4_ core. The enzyme loading capability of TMF was determined by HPLC measurement (Figure 2E). The peak area of the BSA solution with 0.5 μg/μl (a) was 4,273 while the area decreased by 44% to 2,395 after attaching on 8-mg PNIPAM-*b*-PUnAl copolymer grafted magnetic nanoparticles; and, consequently, the concentration of the loading enzyme was 220 μg/mg.

### Digestion Performance Evaluation of TMF Enzymes by Standard Protein

**Figure 3:**
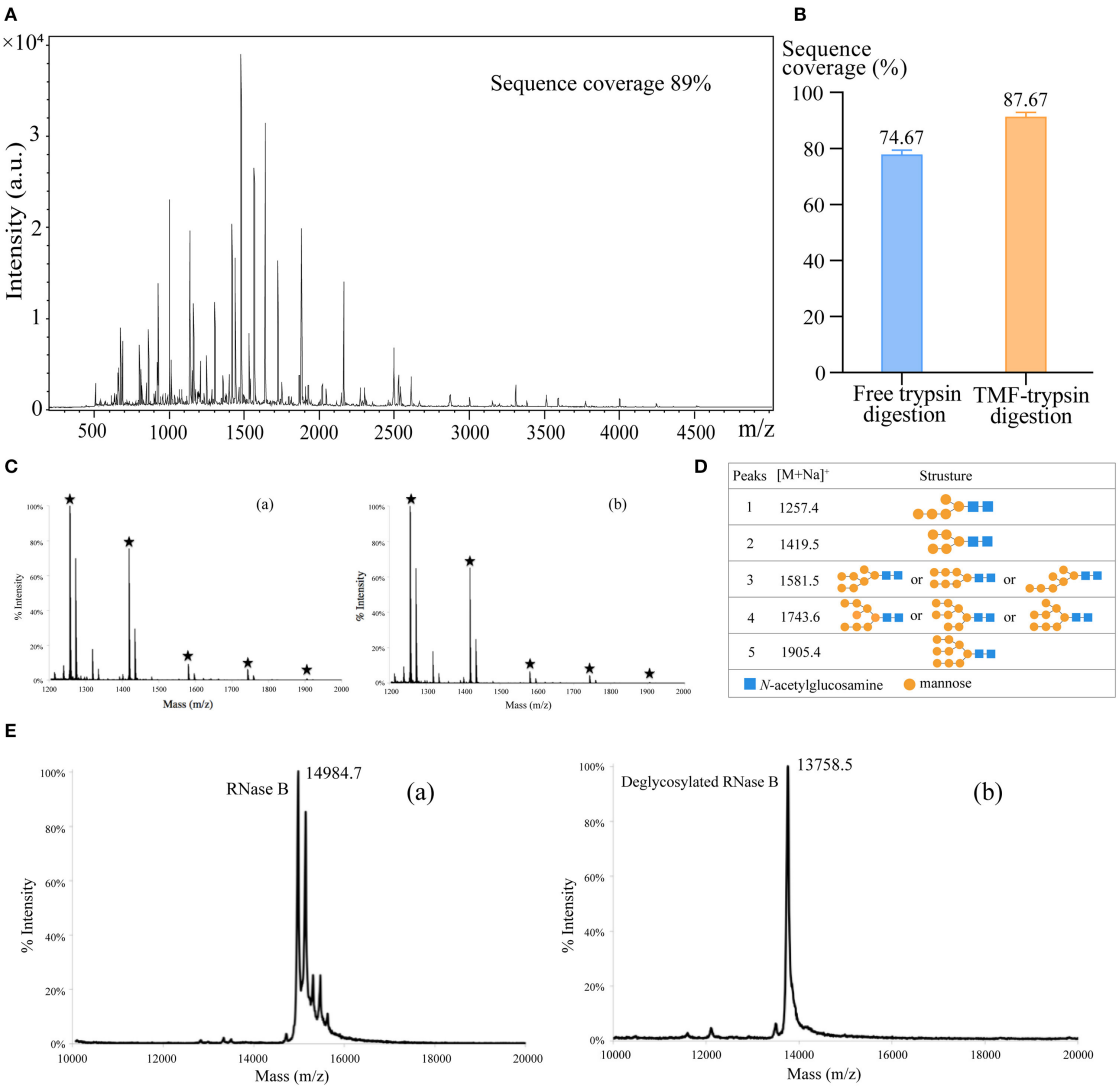
**(A)** The MALDI-TOF-MS spectrum of BSA digests obtained by TMF–trypsin digestion for 3 min. **(B)** Sequence coverages of BSA digested by TMF–trypsin and free trypsin at BSA 10 μg/ml. **(C)** MALDI-TOF-MS spectra of *N*-glycans of RNase B deglycosylated with (a) TMF–PNGase F (room temperature, 3 min) and (b) free PNGase F (16 h, 37°C). **(D)** Observed peaks and proposed structures of *N-*glycans released from RNase B using TMF–PNGase F. **(E)** Spectra of RNase B (a) before and (b) after TMF–PNGase F digestion.

First, we used BSA as a standard protein to examine the performance of TMF-immobilized trypsin. Besides, in-solution free trypsin digestion (37°C, 16 h) was performed as a control. After digestion, TMF–trypsin was recovered by a heating-magnet process, and the supernatant was collected for MALDI-TOF MS analysis. A typical MALDI-TOF-MS spectrum of BSA digests by TMF–trypsin digestion showed 89% peptide coverage (Figure 3A). No obvious peaks exceeding 3,000 m/z could be detected, indicating that TMF–trypsin digestion was completed in such a short time. Moreover, no residual undigested BSA was observed, which demonstrated almost 100% digestion by immobilized trypsin (Supplementary Figure 2). Reducing the amount of BSA to 10 μg, however, can still obtain higher sequence coverage (87.67%) in TMF–trypsin system, and was relatively higher than that of free digestion (Figure 3B). Next, we used RNase B as a model *N-*glycoprotein to examine the performance of the TMF-immobilized PNGase F reagent. RNase B is a small glycoprotein with a molecular weight of ~15 kDa and containing a single *N-*glycosylation site at Asn34, which possesses five to nine mannose residues attached to the chitobiose core (Fu et al., 1994). RNase B was denatured at 95°C for 10 min and then digested with either TMF–PNGase F (room temperature, 3 min) or free PNGase F (37°C, 16 h). Five *N-*glycans [(Man)5–(Man)9] were identified in both conditions with similar signal intensity, indicating the deglycosylation efficiency of the immobilized PNGase F was as good as that of the free PNGase F digestion (Figures 3C,D), but with 320 a fold reduced digestion time. Next, the completeness of deglycosylation using TMF–PNGase F was examined by MALDI-TOF MS in the leaner model. Multiple peaks were detected within the range from 14,984.7 to 15,832.7 Da before digestion of RNase B because of the microheterogeneity of *N-*glycosylation; while only one peak at 13758.5 Da was detected after TMF–PNGase F digestion, indicating complete removal of the *N*-glycans by TMF–PNGase F (Figure 3E). Collectively, the TMF-immobilized enzymes (both trypsin and PNGase F) can efficiently digest (glyco)proteins using significantly reduced time compared to the conventional in-solution digestion system.

### The Combinational Usage of TMF–Trypsin and TMF–PNGase F for Ultrafast Urine *N-*Glycoproteome Analysis

**Figure 4:**
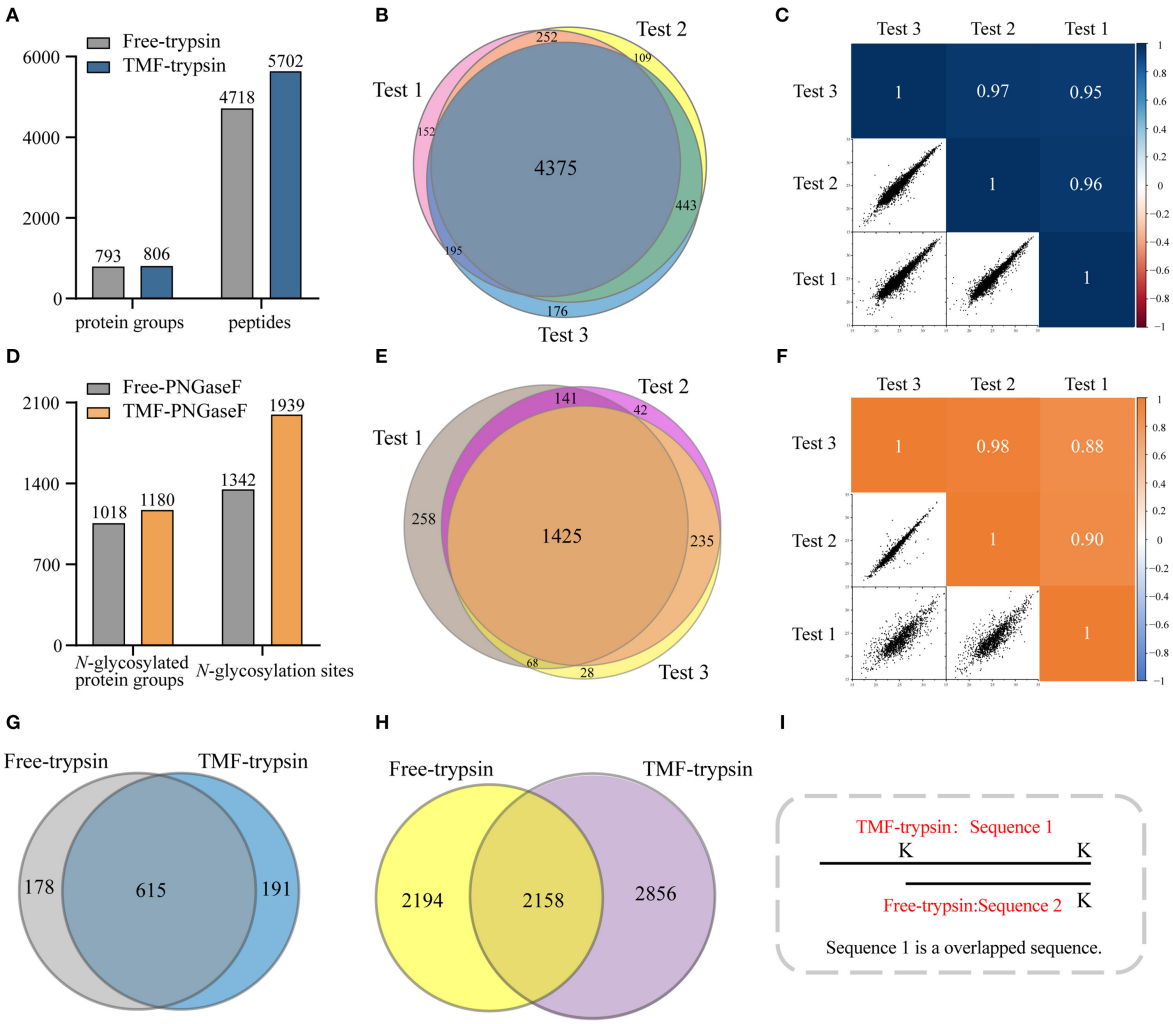
**(A)** Unique peptides and corresponding proteins identified by free trypsin and TMF–trypsin. **(B)** Identification reproducibility evaluation of TMF–trypsin by overlapping the identified peptides of three replicates. **(C)** Quantification reproducibility evaluation of TMF–trypsin. **(D)** Unique *N*-glycosylation sites and corresponding *N*-glycoproteins identified by free PNGase F and TMF–PNGase F. **(E)** Identification reproducibility evaluation of TMF–PNGase F by overlapping the identified peptides of three replicates. **(F)** Quantification reproducibility evaluation of the TMF–PNGase F. **(G)** Overlap of the proteins identified by free-trypsin and TMF–trypsin. **(H)** Overlap of the peptides of the 615 co-identified proteins by free-trypsin and TMF–trypsin. **(I)** Schematic diagram of overlapped sequence caused by missed cleavages.

Long sample processing time and low throughput of conventional in-solution digestion is one of the major limitations for the application of (glyco)proteomic technique in large-scale clinical studies. Using an immobilized enzyme is a promising solution. Unlike other reported immobilized enzymes, TMF-immobilized enzymes have the advantage of homogeneous-phase digestion and heterogeneous separation. Therefore, efficient digestion can be expected by avoiding high-mass transfer resistance in the two-phase digestion system using solid material-immobilized enzymes. Furthermore, TMF-immobilized enzymes can be easily separated from digestion products and recovered through heat-trigged flocculation and magnetic separation. We further evaluated digestion efficiency by sequential application of TMF–trypsin and TMF–PNGase F in urine glycoproteome analysis (Figure 1). After TMF–trypsin digestion of urine proteins, the peptides were subjected to *N-*glycopeptides enrichment by hydrophilic interaction liquid chromatography (HILIC) (Mysling et al., 2010; Cong et al., 2017). The *N*-glycopeptides are further subjected to deglycosylation with TMF–PNGase F in the ${\text{H}}_{2}^{18}$O system. The ^18^O-labeled *N-*glycopeptides were collected for mass spectrometry analysis. With TMF-enzymes, the total sample processing time from urine protein digestion to MS analysis is about 1 h, which is at least 20 times faster than that with in-solution free enzyme digestion. Impressively, both TMF–trypsin and TMF–PNGase F digestion identified more unique proteins and peptides than in-solution digestion (Figures 4A,D). Compared with 793 protein groups and 4,718 peptides identified by free trypsin digestion (Supplementary Tables 1, 2), TMF–trypsin digestion yielded a slight increase in the number of protein groups and peptides, which is 806 and 5,702, respectively (Supplementary Tables 3, 4). TMF–PNGase F also had a better performance, identifying 1,180 *N-*glycosylated protein groups and 1,939 *N*-glycosylation sites (Supplementary Tables 7, 8), while free PNGase F digestion identified 1,018 *N-*glycosylated protein groups and 1,342 *N*-glycosylation sites (Supplementary Tables 5, 6). Furthermore, we conducted three technical replicates to evaluate the reproducibility of the two TMF-immobilized enzymes. In TMF–trypsin digestion, 96% peptides were obtained in at least two tests. Likewise, ~88% of *N*-glycopeptides were identified in at least two tests of TMF–PNGase F digestion (Figures 4B,E). Digestion products of both TMF-immobilized enzymes also have good quantitative reproducibility, with Pearson correlation coefficients higher than 0.95 for peptides and 0.88 for *N*-glycopeptides (Figures 4C,F), demonstrating their capability for actual application in complex samples.

We noticed that relatively more missed cleavage peptides were found in the digestion product of TMF–trypsin, which is disadvantageous for protein quantification. To further evaluate the digestion effect of TMF–trypsin, we analyzed the peptides in the 615 co-identified proteins by the two digestion methods (Figure 4G). Among the 2,856 peptides uniquely identified by TMF-trypsin digestion, 1,089 peptides were with partial sequence overlapping with the corresponding peptides in the free-trypsin digestion product (Figure 4H) because of missed cleavages (Figure 4I). These partial overlapping peptides provide a little contribution to protein identification. However, for the rest of the 1,767 uniquely identified peptides obtained by TMF–trypsin, their amino acid sequences are completely different from those in the digestion product of free-trypsin, which is advantageous in improving the sequence coverage in protein identification. Analysis of amino acids sequence coverage of the co-identified proteins led to similar results. Of the 615 co-identified proteins, ~60% have higher amino acids sequence coverage at the protein level in the TMF–trypsin digestion product. Furthermore, both free trypsin and TMF–trypsin provided a large number of uniquely identified peptides that were not covered by each other. Therefore, combined application of these two digestion methods may lead to increased protein sequence coverage and improved identification reliability, especially for the low abundant proteins, which usually only have a few or no unique peptides identified using only the free-trypsin digestion.

## Conclusion

A novel thermoresponsive magnetic fluid (TMF)-immobilized enzyme (trypsin or PNGase F) for ultrafast and highly efficient proteome digestion and glycopeptide deglycosylation was developed in this study. The high-water dispersity and heat-triggered magnetic separation of TMF-immobilized enzymes make them capable of homogeneous digestion and heterogeneous separation. Therefore, highly efficient and rapid protein digestion and glycopeptide deglycosylation were achieved by avoiding mass transfer resistance in the heterogeneous system, and facile sample recovery was achieved by thermoflocculation and magnetic force. Taking advantage of these unique features, TMF-immobilized enzymes led to the identification of ~2,000 *N*-glycopeptides in human urine sample and, with more than 20 times reduction in sample processing time, demonstrated the potential use of this strategy for fast and high throughput analysis of *N-*glycoproteome in clinical samples.

## Data Availability Statement

The datasets presented in this study can be found in online repositories. The names of the repository/repositories and accession number(s) can be found at: ProteomeXchange Consortium via the PRIDE partner repository with the dataset identifier PXD024639.

## Author Contributions

WQ conceived the project. ZF, TL, and FZ designed the experiments and interpreted data. ZF carried out the synthesis of TMF-immobilized enzymes (trypsin or PNGase F) and performed urinary protein exaction, proteomics experiments, and MS analysis. TL and FZ performed characterization of TMF. ZF and TL performed digestion performance evaluation of TMF-immobilized enzymes. All authors commented on the manuscript.

## Conflict of Interest

The authors declare that the research was conducted in the absence of any commercial or financial relationships that could be construed as a potential conflict of interest.
