# Supplementary figures and images for: An Ultrafast N-Glycoproteome Analysis Method Using Thermoresponsive Magnetic Fluid-Immobilized Enzymes
Source: Front Chem. 2021 Apr 26;9:676100. doi: 10.3389/fchem.2021.676100 (PMC8107388; doi:10.3389/fchem.2021.676100)

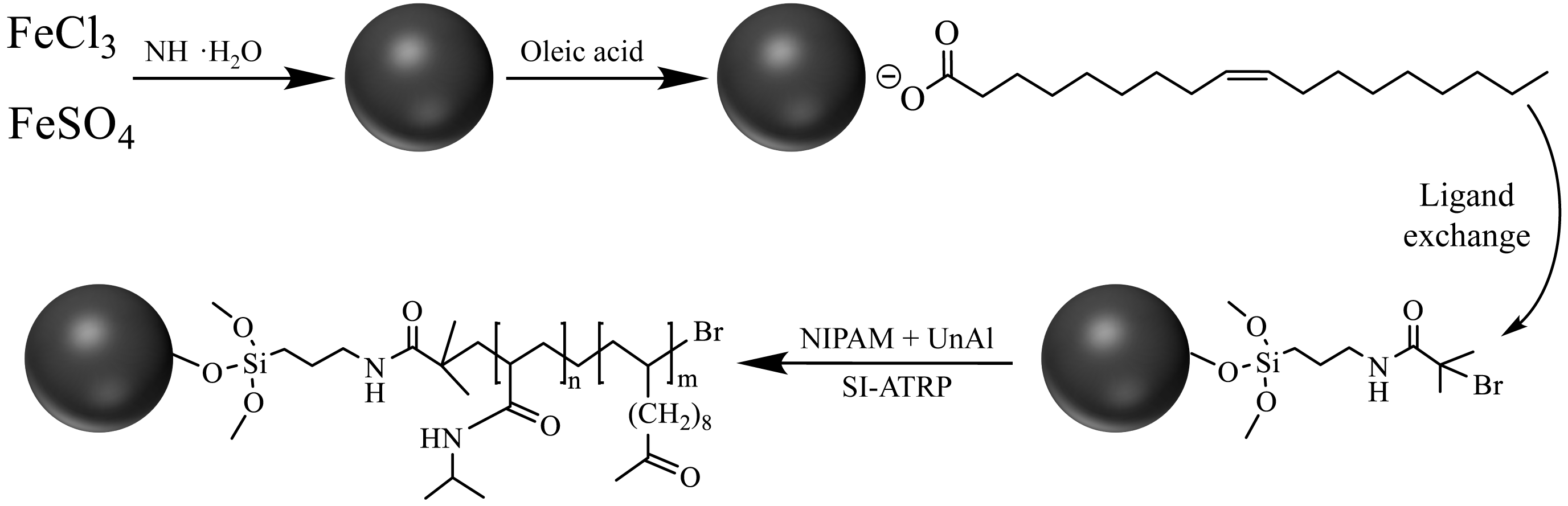

Supplement: Supplementary file 1 [file Image_1.TIF]

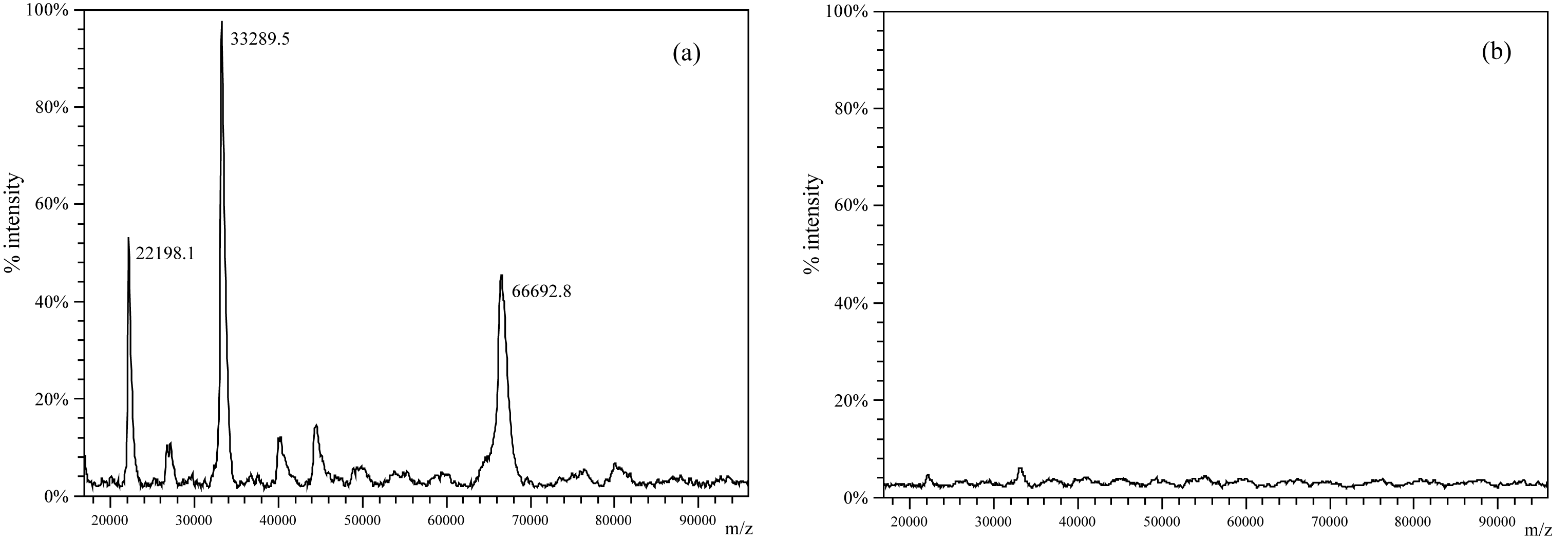

Supplement: Supplementary file 2 [file Image_2.TIF]
